# 

**Published:** 1857-07

**Authors:** 


### CONTENTS.

### ORIGINAL COMMUNICATIONS.

PAGE

ARTICLE I.—An Address in the Atlanta Medical College, before the Æsculapian Society. By A. W. Griggs, M. D., of Newnan, Georgia, June 13th, 1857, ........ 641

ARTICLE II.—Remarks upon Elephantiasis. By V. H. Taliaferro, M. D., Atlanta, Georgia, ........ 647

ARTICLE III.—Supposed Case of Passive Congestion of the Brain, with Effusion. By A. A. Davis, M. D., of Culpepper County, Virginia, ........ 651

### SELECTIONS.

American Medical Association, ........ 654
A Review of the Medical Testimony, in the Case of Charles B. Huntington,.. 675
On "Phantom Tumors" of the Abdomen, ........ 683
Of Sudden Deaths, in the Puerperal State, from Dynamic Lesions of the Nervous Centres, ........ 687
Questionable Advertisements, ........ 692
Somnambulism and Clairvoyance, ........ 694

### EDITORIAL AND MISCELLANEOUS.

To our Friends, ........ 700
Bibliographical, ........ 700
Case of Colica Pictonum, produced by the White Lead Treatment of a Severe Scald, ........ 702
Cure of Vesico-Vaginal Fistula, in a Novel Way, ........ 703
Alum as a Remedy in Croup, ........ 703
Kentucky Giantess, ........ 703
Death from the Inhalation of Amylene, ........ 704
Meteorological Observations for June, 1857, at Atlanta, Ga., ........ 704

